# The Edinburgh Addiction Cohort: recruitment and follow-up of a primary care based sample of injection drug users and non drug-injecting controls

**DOI:** 10.1186/1471-2458-10-101

**Published:** 2010-02-26

**Authors:** John Macleod, Lorraine Copeland, Matthew Hickman, James McKenzie, Jo Kimber, Daniela De Angelis, James R Robertson

**Affiliations:** 1 Department of Social Medicine, University of Bristol, Canynge Hall, 39 Whatley Road, Bristol, BS8 2PS, UK; 2 Muirhouse Medical Group, 1 Muirhouse Avenue, Edinburgh, EH4 4PL, UK; 3 National Centre in HIV Epidemiology and Clinical Research, University of New South Wales, Sydney 2052, Australia; 4 MRC Biostatistics Unit, Institute of Public Health, Robinson Way, Cambridge, CB2 2SR, UK

## Abstract

**Background:**

Injection drug use is an important public health problem. Epidemiological understanding of this problem is incomplete as longitudinal studies in the general population are difficult to undertake. In particular little is known about early life risk factors for later drug injection or about the life course of injection once established including the influence of medical and social interventions.

**Methods:**

Individuals thought to be drug injectors were identified through a single primary medical care facility in Edinburgh between 1980 and 2006 and flagged with the General Registry Office. From October 2005 - October 2007, these cases were traced and invited to undergo interview assessment covering early life experience, substance use, health and social histories. Age and sex matched controls for confirmed cases (alive and dead) were later recruited through the same health facility. Controls for living cases completed the same structured interview schedule. Data were also collected on cases and controls through linkage to routine primary care records, death registrations, hospital contact statistics and police and prison records. All interviews were conducted with the knowledge and permission of the current GP.

**Results:**

The initial cohort size was 814. At start of follow up 227 had died. Of the remaining 587: 20 had no contact details and 5 had embarked from the UK; 40 declined participation; 38 did not respond to invitations; 14 were excluded by their GP on health or social grounds and 22 had their contact details withheld by administrative authorities. 448 were interviewed of whom 16 denied injection and were excluded. Of 191 dead cases with medical records 4 were excluded as their records contained no evidence of injection. 5 interviewed cases died before follow up was concluded though these individuals were counted as "live" cases. 1 control per case (dead and alive) was recruited. Linkage to Scottish Morbidity Records data (available from 1981 onwards) on general acute inpatient and day cases, mental health inpatient and day cases and cancer was provided by Information Services, NHS Scotland, for all cases interviewed and all dead cases. The Scottish Prison Service provided records for 198 (46%) of cases interviewed, 48 cases not interviewed and 34 (18%) of dead cases. For a sub-sample of 100 interviewees a search of the Lothian and Borders police database was made for official criminal records and 94 had criminal records. Data linkage for controls is ongoing.

**Conclusions:**

Injecting drug users recruited from a community setting can be successfully followed-up through interviews and record linkage. Information from injecting cases is being analysed in terms of injecting patterns and possible influences on these. Comparisons between cases and controls will allow identification of possibly modifiable early life risk factors for drug injection and will also clarify the burden of disease associated with injection and the influence on this of different health and social interventions.

## Background

Illicit injection drug use, particularly of opiates, emerged as an important public health problem in the second half of the 20^th ^century [1,2]. Injection drug users (IDU) experience increased risk of morbidity and mortality mainly related to viral and bacterial infections, accidental overdose, and injection related arteriovenous occlusions [3,4]. Injection drug use also has social costs, particularly from associated criminality, for injectors, their families and the community [5]. There is also evidence that blood borne infections acquired by injectors may be transmitted to their non-injecting sexual partners [6].

There is limited evidence of effective primary or secondary prevention of injection drug use [7]. The most evaluated secondary prevention intervention is the prescribing of non-injected substitute drugs, in particular oral methadone [8]. There is evidence that this can reduce injection frequency and illicit drug consumption and is also associated with reductions in morbidity and mortality [9-12]. It is uncertain whether and how drug treatment modifies overall duration of drug dependence and injecting. Existing evidence is mixed; some longitudinal evidence suggests that methadone treatment is associated with shorter injecting careers [13] whilst other evidence suggests no beneficial effect of treatment on mortality amongst recent onset injectors in the community [14].

Aside from oral substitution treatment, injectable substitutes have also been evaluated [15], as have various approaches to supporting detoxification either as an inpatient or in the community [16]. Outside the health sphere the main interventions aimed at injection drug users are those delivered within the criminal justice system in the form of custodial and non-custodial sentences that may include an element of drug treatment but these have been less rigorously evaluated [17,18].

There is currently limited evidence on the effect of all these interventions when they are delivered in naturalistic settings outside of study conditions. Similarly the epidemiological understanding of risk and protective factors, natural history and outcomes of injecting that might inform the development of more effective interventions is also deficient. There are several reasons for this deficiency. Injection drug use is an illegal, clandestine activity undertaken by individuals that often lead highly marginalised lives and typically experience a high degree of socio-environmental disadvantage making the recruitment of representative samples and follow-up difficult [19]. Indirect estimates of drug injection prevalence in some UK cities suggest it is not uncommon [20,21], at 1-2% of younger adults in most UK cities. However, attempts to sample IDU through household surveys fail because of numerous selection biases [22,23]. Epidemiological studies of drug injectors have frequently been based on individuals recruited from specialist treatment settings [24-27], or through a form of snowball sampling and community recruitment [28-31]. Such individuals are often recruited at a relatively late point in their injecting career and may be atypical. Moreover, many studies of injectors are cross-sectional and therefore limited in their ability to inform causal hypotheses. Prospective, general population based studies of injectors are extremely rare since injectors are less likely to be recruited or retained in such investigations. Another substantial problem facing observational studies of drug injectors is that of confounding. Distinguishing genuine causes and consequences of injection from factors that are mainly markers of the disadvantage injection drug use is typically associated with is a considerable challenge.

Nonetheless, Injection drug users often have relatively high levels of contact with various services and official bodies who may record and hold data relating to these contacts. These routine data may be a valuable source of information relevant to epidemiological studies.

In the UK, most medical treatment for injection drug users is delivered in primary care. Primary care based treatment for injectors has a longer history in South East Scotland than in much of the UK because of local need to respond to an epidemic of HIV infection amongst drug injectors in the 1980s [32]. The Edinburgh Addiction Cohort (EAC) is a community based, open cohort of opiate injectors presenting to a single primary medical care facility in North West Edinburgh between 1980 and 2006 [33]. Cohort members have been followed up since recruitment through routine data sources, including their primary care records and through personal interview. More recently, a cohort of age and sex matched non-injecting controls has been recruited through the same primary medical care facility. Information on these controls is collected from the same sources as with cases. Cases and controls will be used to investigate questions around the aetiology of injection drug use in terms of risk and protective factors; disease course and the influence on this of exposure to medical and criminal justice interventions and other factors; and outcomes. Comparisons with non-injecting controls from the same population as cases also allows consideration of the issue of socio-economic confounding.

## Methods

### Data collection instruments

Cases and controls completed an interviewer-administered structured questionnaire (Additional file 1) developed by the research team during the course of a Chief Scientist Office-funded pilot study (May 2004 - March 2005). Questionnaire domains included early life family circumstances, social environment and experience of various types of adversity, education and employment, licit and illicit drug use, contact with primary and secondary medical care services and treatments received, forensic history, relationships and children, sources of income, homelessness and housing, physical and mental health. Participant recall of historical events was facilitated using the life-grid approach [34] whereby events are related to personally significant events that can be anchored to a historical timescale (for example the deaths of famous people, particular sporting occasions etc). Questionnaires also incorporated standard instruments including the Audit Scale for Alcohol Dependence, the Fagerstrom Test for Nicotine Dependency, the Hospital Anxiety and Depression Scale and the EQ-5D Health-Related Quality of Life. J McK, an experienced community psychiatric nurse, undertook most of the interviews; JR, MH and JM carried out a smaller number.

### Estimating duration of injecting career

Amongst drug injectors the total number of years injecting was derived by subtracting the year of injection initiation from the year of last cessation or year of follow-up if still injecting. In order to allow for periods of injection cessation in this calculation we asked participants for each year after they started injecting whether he/she had injected in a given year (yes/no), and if so, whether he/she had ceased injecting for three months or longer in that year (yes/no), and number of periods of injecting cessation (range 1-3 times). "Inject time" was calculated based on the number of cessations and the assumption that any given cessation was of three months duration. Thus, for any given year the value was set at 0 for those who did not inject in that year; 0.25 if an individual had three 3 month cessations; 0.5 if an individual had 2 cessations, 0.75 if an individual had 1 cessation and 1 if the individual injected throughout the year. "Inject time" was then aggregated across years to calculate the number and duration of episodes of injecting and cessation based on the following rules: (1) all consecutive whole years of injecting form a single period of injecting; (2) all part years of injecting when they either preceded or followed by a full year of injecting are aggregated; (3) two consecutive years with part of the year injecting are aggregated and any third year begins a new episode [27] Clearly, inject time is right truncated as some of the participants are still injecting, and some that report not injecting at the time of follow-up may subsequently relapse. The nature of the data mean that the appropriate analysis of inject time in relation to survival and cessation must be based on methods and statistical models that handle discrete time periods (i.e. aggregated time periods and the number of events that occur within them) rather than continuous time, and that to measure duration of injecting a definition of "final cessation" is required [35,36].

A template for extracting relevant data from primary care records (Additional file 2) was also used, which covered areas such as problems in childhood, illicit drug use and substitution treatment, physical/mental health problems and blood borne virus data. This template was also used for extracting information from the primary care records of dead cohort members, along with a supplementary data sheet relating to drug and alcohol use in the year prior to death and scrutiny of death certificates for relevant information.

### External data sources

In addition to interview data and primary care records, the individuals were linked to other external data sources.

1. Scottish Morbidity Register (SMR) data - in order to identify cohort members that since 1981 have been admitted to general acute hospital as inpatient or day cases or mental health ward/hospital as inpatient and day cases, or had cancer registration.

2. The Scottish Prison Service (SPS) used SPS Prisoner Records System Version 2 to search their database (initially electronic in 1996 and revised in 2004) for cases' prison records and provided data on types of crimes committed and sentences given to our cohort members.

3. Lothian & Borders Police provided criminal record systems containing crimes and disposals for a sample of 100 interviewees and 19 dead cases. Searches for the full number of cohort members could not be carried out due to lack of police resources.

4. General Register Office (GRO) for Scotland provided the study with tracing data and death certificates.

In each case the relevant data custodian was provided with identifier information on cohort members (for SMR data and data from the GRO this was name, sex, date of birth and National Health Service (NHS) number; for police and prison data this was name, sex and date of birth) to allow matching. Only exact matches on these fields were accepted. For SMR, Prison and Police data a single matched file of information up to the date of matching was provided to the study team. For death certificates information is provided on a rolling basis as deaths are registered.

Initially, it was also our intention to attempt individual linkage to files held by the Edinburgh Social Work Department. This subsequently proved impossible. Though the Department were prepared to confirm whether individuals had a Social Work case file, they would not allow access to information contained in that file due to concerns around confidential third party information.

### Recruitment of cases

Cases were recruited between 1980 and 2006 when they presented at Muirhouse Medical Group with a history of injection drug use. Throughout the recruitment period a dedicated research project worker undertook regular note reviews in an attempt to ensure complete ascertainment of presenting cases. The project worker entered cases on a clinical database. Cases were "flagged" with the GRO for Scotland to allow tracing, the collection of death certificates and possible linkage to the Scottish Morbidity Register (SMR). The GRO did not require individual consent for such flagging and all cases were therefore flagged.

From 2005, when the present study commenced, individual consent was sought from all living cohort members contacted for follow up for linkage to the medical and social administrative databases described above at the time of interview. All individuals interviewed gave such consent. The follow up study attempted to contact all surviving cohort members between October 2005 and September 2007. For those no longer practice patients, tracing was carried out via GRO, Practitioner Services and Primary Care Trusts in order to establish details of patients' current registered General Practitioner (GP). Where possible GPs were approached directly, given study information and asked to provide patient contact details. In some cases direct GP contact was not possible as current GP was not provided by the relevant Director of Public Health. In other cases some GPs were not prepared to provide patient contact details. In both these latter scenarios GPs and Directors of Public Health were asked to forward a request for participation in a follow-up interview to cohort members. Where contact details were available these requests were sent directly to cohort members by the study team. Cases agreeing to be interviewed were invited to select their preferred venue from their own home, a site provided by the researcher (generally a local practice) or an alternative venue such as a café or a friend's home. 3 cases were hospital in-patients at the time of interview.

### Recruitment of controls

Controls were recruited from the current Muirhouse practice list (approximately 11,000). Potential controls of the same sex as cases and with age +/- two years of the age of cases but with no history of injection drug use were identified on the practice list. Controls were recruited between January 2008 and July 2009. During this period two of the research team (JRR and JMcK) noted all potential controls amongst patients attending the practice to see a GP or other health professional each day. These patients were then approached (by JMcK or JRR), informed of the study and invited to participate. This process continued until a sex and age (+/- two years) control was recruited for each case. Those who agree gave signed consent and were interviewed at a time and location of their choice. Controls for living cases were interviewed using the same schedule as cases, the same data items were extracted from their patient notes, and (subject to consent) they were flagged with the General Register Office for Scotland and the Office of National Statistics and the Scottish and English prison services. In addition, controls were asked for consent to linkage with police computer and SMR 1 and 4 databases. Controls for dead cases underwent all the above assessments (with appropriate consent) other than personal interview. Controls reporting previously unobserved or unrecorded injecting drug use, were eligible to be included as a case, with the reasons for their unobserved status assessed however no controls reported injection drug use. Non-injection use of illicit drugs does not effect control eligibility; this possible explanatory factor is measured in both cases and controls so that its influence can be investigated.

### Ethical approval

For the cases study, ethical approval was obtained from the Lothian Research Ethics Committee 04 (LREC/2003/7/12). and the same Committee granted ethical approval for the subsequent controls study (LREC/07/S1104/20.

## Results

### Recruitment of cases and controls

**Figure 1:**
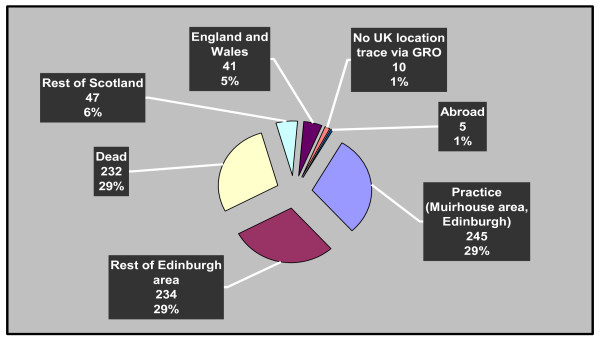
**Geographic distribution of the EAC cohort including the 20 misclassified cases (n = 814)**. "pie diagram" showing the distribution of cohort members at follow up.

Figure 1 illustrates the geographic distribution of the cohort at the close of case recruitment in October 2007. While many cases still resided in the Edinburgh and Lothians area, a significant number were now located in different parts of the UK. 814 individuals (555 males) were originally included in the EAC and flagged with the GRO. At the start of the follow-up period 227 of these had been notified as dead. We attempted to trace and recruit the remaining 587 as described above. 20 were untraceable and 5 had embarked from the UK. Of the remaining 562, 22 had their contact details with-held by either their GP or by local NHS administrative authorities (generally the Director of Public Health) in most cases the individual making this decision (that was generally justified on grounds of confidentiality) offered to pass on study details. These offers were always taken up though we had no means of verifying which cases actually received forwarded information and no cases were successfully recruited through this mechanism. 40 individuals responded to study invitations declining to participate, 38 individuals did not respond to repeated contact attempts including home visits. Our protocol allowed GPs to exclude individuals from the study where they felt that the individual was either too unwell to participate or where an invitation might cause distress. 14 individuals were excluded in this way.

**Figure 2:**
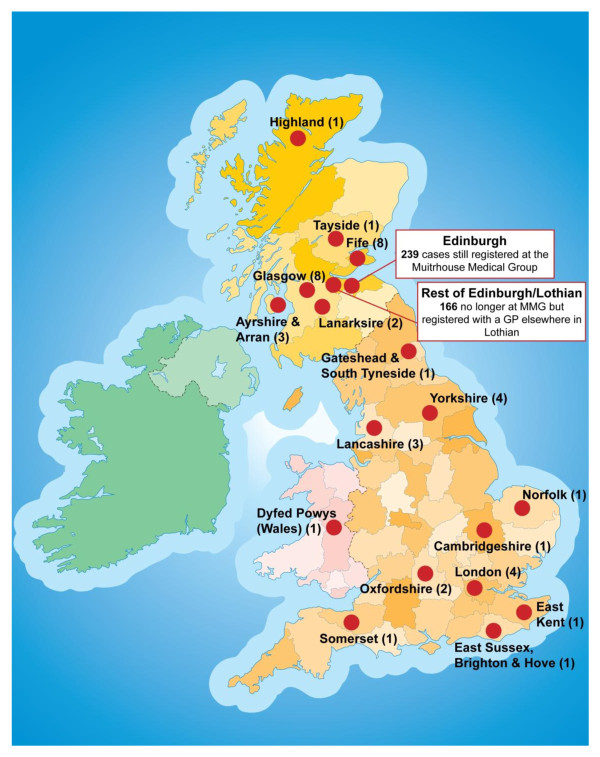
**Geographic location of follow-up interviews (n = 448)**. UK map showing location of follow-up interviews

448 individuals were interviewed (85% of the 526 with whom direct contact by the study team was attempted). The geographic distribution of interviews in the UK is shown on Figure 2. All those interviewed gave consent for all record linkage. 42 individuals interviewed denied ever injecting drugs. In these cases corroborating information was sought from other sources. In 26 cases reports of injection were previously documented in their primary care records. Following these disclosures the 191 available notes of dead cases were searched and in 4 there was no corroborating evidence that the individual had ever been an injector. These 4 were also excluded leaving 432 live and 223 dead cases. 5 cases died during the follow-up period after having been interviewed but for control selection purposes these were still considered "live" cases as interview data were available.

The characteristics of cases at recruitment are shown in Table 1. Three-quarters of cohort participants were born in Edinburgh. Their average age at their first injection was 19.9 years and at study recruitment was 26.7 years. Half were recruited within five years of injection onset.

**Table 1:** Cohort characteristics at recruitment

Characteristic	N		
Male (%)	794		543 (68.4)
Born in Edinburgh (%)	794		530 (66.7)
Mean age (SD, min-max) at first injection	606*		19.9 (5.1, 11-41)
Mean age (SD, min-max) at recruitment	606		26.7 (6.3, 16-52)
Mean years (SD, min-max) first injection to recruitment	606		6.9 (5.7, 0-28)
Recruited within 5 years of injection onset (%)	606		306 (50.5)

* Only 606 cohort members had information on age of first injection

The characteristics of the cohort at follow-up are shown in Table 2. More than a quarter of cases (29%) were deceased by the end of follow-up. Mean years of follow-up (i.e. year of follow-up/death minus year of recruitment) for cases was 10.2 years (SD 6.7, range < 1-25). Of those interviewed (54%), almost one third (31%) reported current injecting at follow-up, more than two thirds were in opiate substitution treatment (70%), and less than one in five had ceased injecting and were opioid-free. Almost all were smokers (93%) and one in five were also problem drinkers (20%).

**Table 2:** Follow-up status of cases and comparison of the current health status of cases and controls

Follow-up status of cases	N = 794 (%)	% Interviewed (N = 432)
Deceased	228 (28.7)	0.1^a^
Interviewed	432 (54.4)	100
Case notes available	654 (82.2)	100
Lost to follow-up	139 (17.5)	-
Mean years follow-up	10.2 (SD 6.8, range < 1-25)	
		
Current injector	135	31.3
Current OST	302	70.0
Opiate free^1^	75	17.4
		
**Controls vs. cases health status**	**% Controls (n = 432)**	**% Cases (n = 432)**
Smoker	255 (59.0)	403 (93.2)*
High risk alcohol use^2^	60 (13.9)	87 (20.1)#
Anxious^3^	87 (20.1)	209 (48.3)*
Depressed^4^	49 (11.3)	114 (26.4)*
Mean subjective QoL (SD)^5^	63.8 (22.7)	50.3( 23.6)^

Notes: a: Five interviewees subsequently died before the end of the follow-up period. 1. Not in OST or injecting illicit opiates 2. Based on AUDIT score of ≥ 16 which indicates high risk or harmful drinking in the past year [48] 3. Based on a HADS anxiety subscale score ≥ 11 indicating caseness [49] 4. Based on a HADS depression subscale score ≥ 11 indicating caseness [49] 5. Mean EqVAS score, where a score of 0 is worst imaginable health state and of 100 is best imaginable health state[50]. Chi square test for difference: # p < 0.01, *p > 0.001, ^ t-test for equality of means: p < 0.001.

The study has recruited one control per case (i.e. 432 "living" controls and 223 "dead" controls). Comparison between cases and controls in terms of tobacco and alcohol use, psychological health status and quality of life are presented in table 2. Controls showed lower prevalence of smoking and problem drinking than cases, and had better psychological health and quality of life. Detailed comparisons between cases and controls are not the subject of this paper and will be reported elsewhere. 19 potential controls were invited but declined to participate in the study and two "living" controls agreed to be interviewed but declined consent for record linkage.

## Discussion

### Response rate

We interviewed 85% of cohort members who had the opportunity to respond to a study invitation (though 16 of these individuals were subsequently excluded as cases on the basis of interview information). A very small proportion of individuals invited to be controls declined (see above). Despite our relatively high response rate amongst cases it is possible that there may be systematic differences between responders, non-responders and those declining participation. Non-response may have occurred in the context of more chaotic lifestyles with frequent address changes thus our follow-up may have been biased towards less problematic individuals. Conversely some non-response and particularly some instances where individuals actively declined participation may have reflected situations where the individual had moved on from a drug injecting lifestyle. In this situation more problematic individuals may have been overrepresented amongst interviewed cases. Systematic differences between traced and non-traced cohort members are also possible. For example in some cases failure of tracing (as distinct from non-response or refusal) might also have reflected a more chaotic lifestyle with failure to register with a GP. All prospective studies are prone to bias resulting from non-response and missing information and the possible influence of such bias will be discussed in relation to specific analyses when these are presented. In this regard it is worth noting that our success in follow-up was higher than that in several other UK studies with a considerably shorter follow-up interval [25,31,37].

### Issues related to the questionnaire instrument

The questionnaires used in the structured interviews included standard instruments on tobacco and alcohol use, psychological health and quality of life as described above. A previously validated questionnaire covering all the domains of interest in our study was not available therefore a study specific questionnaire was developed. In developing the questionnaire we sought advice from various experts. Most of these were senior investigators in the addictions field with a particular interest in the epidemiology of IDU who provided guidance on questionnaire content based on experience in their own studies and the instruments they had developed for these. We also sought additional expert advice on use of the "life grid" interview method to measure early life experience in adults and advice on specific issues related to the measurement of criminal histories. The questionnaire was tested for comprehensibility and other aspects of performance during pilot work and refined where necessary. The expert input we received provided reassurance regarding face validity. No further validation of the instrument was possible prior to the study indeed further validation would have been difficult due to lack of a gold standard for comparison. Triangulation of some questionnaire measures with measures obtained through linkage allowed some further validation as discussed below. A copy of the questionnaire is included as an appendix to allow readers to judge its probable validity.

### Success of linkage

The main general limitations of linkage are that linkage may fail due to unreliability of linking identifiers; a particular data item may not be present in the linked data set (which may or may not reflect issues of data completeness) and that the validity of the measures in the linked data set may be compromised - basically because they were not collected with the research purpose for which they are now being used in mind.

With these caveats in mind the success of linkage in providing comparison records across different data sources is summarised in table 3. 222 of 223 dead cases had death certificate information (one case died abroad and whilst the death was notified no death certificate was available) and 182 (82%) had full primary care records available to the study. All but one set of records not available had been destroyed, in a single case the participant's last GP declined to provide a copy.

**Table 3:** Comparison records available across different data sources (NB. lack of comparison record does not necessarily imply failure of linkage as some individuals will not have experienced record generating events in relation to all data sources)

Data source	Records attempted to link	Linkage successful
Scottish Morbidity Register (live confirmed cases)	432	432
Primary care records (live confirmed cases)	432	432
Primary care records (dead confirmed cases)	223	182
General Register Office for Scotland (dead confirmed cases)	223	222
Scottish Prison Service (live confirmed cases)	432	198
Lothian and Borders Police (subset of live confirmed cases)	100	94

All 432 interviewed live cases had both primary care records and were successfully linked to the Scottish Morbidity Register. We were unable to access primary care records or SMR records on live cases not interviewed, as we had no opportunity to obtain consent. The Scottish Prison Service (SPS) did not require individual consent for record linkage so linkage was attempted on the full original cohort of 814; records were available on 288. 206 of these had been interviewed though 8 of these were individuals who denied ever injecting and were thus removed from cases, 48 were traced but not interviewed and 34 were dead cases. Lothian and Borders Police required modification of our original consent form before undertaking linkage. Linkage was attempted on the first 100 individuals who consented to this to assess the likely yield. 94 of these individuals had police records though one denied injecting and was removed from cases.

We are in the process of undertaking detailed comparisons of data available on study participants from different sources and these will be reported separately. For most data items the most complete source data are those from interviews of live cases. Recall and social desirability bias may influence the validity and reliability of interview information (see below). To an extent this influence can be assessed through triangulation with data on an equivalent measure from other sources though there are limitations on the scope of this. The medical records of all individuals interviewed are available for comparison and corroboration of medical history. 398 individuals reported ever receipt of a substitute prescription and for 396 of these this was recorded in their primary care notes - it is possible that notes may be incomplete. 324 individuals interviewed reported referral to specialist drug treatment services though 387 had evidence of such referral in their notes. The discrepancy may reflect recall bias or the fact that some individuals may have never attended a specialist appointment and/or been aware that a referral had been made. Levels of apparent agreement between interview information and primary care records for a selection of data items are presented in table 4. Detailed comparison of secondary care contact reported at interview with that recorded in the SMR has not yet been undertaken.

**Table 4:** Comparison of agreement across different data sources of selected data items

Variable	Reported at interview (n = 432)	Noted in primary care records (n = 432)	Agreement (%)
Ever on OST	398	396	99%
Ever injected*	406	393	97%
Ever referred to specialist drug treatment service	324	387	84%
Ever overdosed and been seen by a doctor	214	152	71%
Ever seen a doctor regarding alcohol problems	72	71	99%
Current smoker	403	281	70%
Currently medically unfit for work	300	324	93%

* See text for discussion

321 of the 432 cases interviewed reported a history of past imprisonment and 195 of these had SPS prison records. Individuals incarcerated outside of Scotland or before 1996 would not have an SPS record which may explain some of the apparent discrepancy. 3 individuals with SPS records denied ever having being imprisoned at interview. This could reflect either biased reporting or mistaken identity in linkage. Cases were asked about childhood rather than adulthood police contact at interview though lifetime police records were linked as these also contain information on imprisonment. 58 individuals reported childhood (i.e. up to age 16) police contact and in 56 cases their police records corroborated this. Police contact outside of the Lothian and Borders region would not necessarily be reliably recorded in the Lothian and Borders database.

### Potential biases

Potential biases in our study and their possible influence are summarised in table 5. A fundamental bias that it is impossible for us to avoid relates to the fact that our cases were derived from individuals attending a health facility and disclosing injection drug use. Study mechanisms should have ensured that our ascertainment of these individuals was reasonably complete. It is, however, possible that some individuals disclosed injection drug use that was never recorded in their notes and that these cases were not identified by the study. It is likely that cases who were identified were an incomplete sample of drug injectors in the community. Anecdotal evidence suggests that a high proportion of injectors in the local community sought care in relation to their drug use at Muirhouse mainly because the practice had a reputation for a sympathetic attitude towards drug users and a relative readiness to prescribe substitute drugs. Because of these factors it is likely that our cohort represented a high proportion of problem opiate injectors in the community. Occasional injectors and those able to "control" their use would have had less reason to identify themselves to the practice as the potential stigma attached to this may have been perceived to outweigh any possible benefit. Such injectors are therefore likely to have been under-represented in our cohort. 40 out of 472 (8%) eligible living cases declined to participate in follow up (28 male, 12 female, mean age 40 years). The majority (80%) of these individuals were no longer patients at Muirhouse and, since they had declined consent, limited information was available on which to base a comparison with cases who did participate. Most individuals declining participation in follow up gave no reason for this decision. Of the two that did both stated that injection drug use was a part of their life that they had "moved on" from. It is possible that this reason was more widespread amongst those declining follow up which may have biased the sample towards individuals with more enduring drug problems, however since the proportion declining follow up was small overall such bias is unlikely to have been a substantial influence.

**Table 5:** Potential biases, their influence and how this may be mitigated

Potential source of bias	Impact of this bias	Possible strategies to minimise this and other relevant considerations
Selection bias with regard to initial case ascertainment since cases were all service users and IDU were not selected at onset of injecting	Causes, consequences, natural history and duration of IDU amongst injectors who do not present to services may be different	Impossible to avoid however likely to be less of an issue than in studies where cases are recruited from specialist clinics as this involves additional level of selection. In addition, time from onset of injecting to recruitment in this study shorter than in most other cohorts.
Survival bias with regard to case follow-up	Patterns of association between the factors under study may have been different amongst living compared to dead cohort members	Information on most factors of interest was available through record linkage on both living and dead cohort members
Selection bias with regard to case follow-up. Cases successfully followed up were willing to be interviewed. Unwillingness to be interviewed may have reflected either more chaotic current circumstances or a reluctance to discuss long resolved drug problems.	Patterns of association between the factors under study may be different amongst those lost to follow-up. Outcomes of IDU may have either been over or underestimated	Impossible to avoid though loss to follow-up was relatively low and much was due to structural factors (e.g. GP unwillingness to recruit) unlikely to be related to participant characteristics
Selection bias with regard to control recruitment as controls were all attending a health facility	If controls were more likely to have health problems than the general population this may have diluted associations between some risk factors and outcomes in case-control comparisons	The majority of the population use primary care services relatively regularly often for reasons unrelated to a significant health problem and any "unhealthy participant" effect is therefore likely to be small
Selection bias with regard to control recruitment as controls may not have been a representative sample of service users	Potential controls declining recruitment may have been different from those agreeing with regard to the factors under study	Consecutive eligible service users were approached during control recruitment. Only 3% declined suggesting substantial bias is unlikely
Selection bias with regard to control recruitment as controls were not recruited at the same time as cases	To be recruited controls must be alive and resident in the practice area. Despite age and sex matching this may have introduced bias.	Impossible to avoid as control selection from reconstructed historical practice list was unfeasible (see text). Impact may not have been substantial since healthier controls would be both more likely to be living but may also have been more likely to leave practice area. These influences would tend to cancel each other out in terms of resulting bias.
Social desirability bias in relation to interview measures	Cases may have been more likely to disclose drug use and other socially sensitive behaviours and exposures leading to overestimation of the association between these factors and IDU	Assurances of confidentiality and good relationship with practice team should have mitigated this. Where possible objective corroboration with measures collected through linkage was sought
Recall bias in relation to interview measures	Case recall of some early life exposures may have been influenced by their own beliefs around causes of IDU leading to overestimation of the association between these factors and IDU. Substance use may also have impaired case recall of previous exposures leading to underestimation of the association between these factors and IDU.	Use of the life-grid approach should have mitigated this. Where possible objective corroboration with measures collected through linkage was sought
Strong association between disadvantage and IDU may lead to confounding of case-control comparisons	Some apparent effects of both IDU itself and possible risk factors may in reality be effects of other correlates of disadvantage	Recruitment of controls from the same community as cases should mitigate any bias of this type and measurement of individual social position allows further adjustment

There are also potential selection biases between cases and controls, primarily as the controls are living and still patients at Muirhouse. We determined through a pilot study that it was not feasible to reconstruct the historical practice list back to the time of recruitment of all the cases, thereby allowing selection of retrospective controls and avoiding this bias. Further, we felt that the selection of historical controls no longer in contact with the practice would introduce practical and ethical problems of follow-up since unlike cases these individuals had not consented to tracing. Even if ethical approval for tracing were granted we felt that response rates amongst these controls (given our experience of the administrative hurdles during follow-up of the cases) would probably be low. Information collected at interview is subject to the bias normally associated with self-report in particular social desirability and recall biases. It is possible that these may have differed between cases and controls. For example cases may be more likely to report (rather than experience) adverse childhood exposures and may also be less influenced by social desirability considerations in relation to their reports of drug use and other socially disapproved behaviours. Our use of the life-grid interview method, assurances around confidentiality and the good relationship between the practice and its patients should mitigate these considerations to an extent. Broadly, controls *are *comparable to the cases i.e. representative of the population that generated the cases which is the critical issue with regard to validity of case control comparisons [38]. 19 potential controls (16 male, 3 female, mean age 43, 3% of the total 655 required) declined participation. Again since these individuals had not consented to study participation no comparison was possible between them and those who did consent in terms of the variables under study. Amongst those who gave a reason for non-participation, inconvenience was a common explanation cited. Given that the proportion of potential controls declining participation was very small it seems unlikely that this would have introduced substantial bias.

### Natural History

Population surveys and longitudinal studies of IDUs give two very different pictures of injecting duration, stemming partly from distinct selection biases. Population surveys under-represent current IDUs; and longitudinal surveys tend to under-represent non-dependent IDUs and those with short duration. Thus psychiatric morbidity surveys in UK [39] and US [40] suggest that of people who report ever using heroin only 25 to 35% were dependent and used for sustained periods of time, and therefore that the average duration across all IDUs and heroin users estimated from these surveys would be comparatively short. It is possible to obtain unbiased estimates of duration of use from biased population survey data, the ability to do this relies on the availability of additional information difficult to collect [41,42].

In contrast, many longitudinal studies of IDUs emphasise the potentially long duration and high mortality associated with injecting heroin use. For example, a recent analysis of heroin trends in Switzerland [43] estimated a mean duration of 25 years. Further, most models of injecting assume a single cessation episode and a continuous duration, which we know is not realistic as injecting drug use typically is a chronic relapsing condition.

Richer descriptions of injecting duration are becoming available from a limited number of cohorts. For example, analyses of the California Addiction Cohort suggest that for active participants who had injected within five years of the previous interview approximately 15% were abstinent ten years later; whereas of those who had not injected for longer than five years approximately 75% remained abstinent 10 years later [44]. Participants from the Amsterdam Addiction Cohort reported over 2 periods of injecting per person (range 1 to 8) and over 1100 periods of cessation during an average 9 year follow-up [45]. The mean duration of cessation was 13 months; with approximately half relapsing within the year and 85% by five years. In addition, an analysis of the ALIVE cohort from Baltimore reported that approximately 70% of IDUs ceased drug use after an average of 4 years, but with 75% relapsing within an average of one year [46].

Information on cessation also is provided by observational cohort studies that recruit drug users from specialist treatment. For example, the National Treatment Outcome Research Study (NTORS) reported that five years after recruitment, 25-33% of the sample were no longer regular heroin and/or injecting drug users [25,47]. The Drug Outcome Research Study (DORIS) in Scotland reported that 8% of subjects were entirely "drug (i.e. opiate) free" after 33 months follow-up; but if licit opiate substitution therapy was excluded from the definition of opiate use then approximately 13% were "abstinent" at follow-up [26]. Using data from these cohorts it is not possible to estimate the long term impact of treatment on injecting duration, partly because follow-up is too short but also because selection is conditional on exposure to treatment.

Similarly, long term cohorts such as the ALIVE and Amsterdam cohorts may suffer from selection biases since participants were recruited on average later than 10 years after onset of injecting [28,45]. This may have a strong influence on the estimation of the cessation rates as considerable amount (40 to 60%) of injecting history is unobserved and, particularly, those IDUs with short injecting duration will be considerably under-represented.

Data from EAC will provide additional insight on injecting natural history. The EAC cohort has, on average, less unobserved periods of injecting before recruitment than other current cohorts, although it also has less frequent follow-up. The challenge of new analyses of natural history and injecting duration will be to: a) exploit fully the information from the follow-up data, which are interval censored data i.e. only the current status (injecting or not) of each individual is known, rather than the precise date of ceasing injection and relapsing; b) address the potential multiple biases affecting the data, including recruitment (or left truncation) and follow-up biases. Left truncation refers to fact that subjects were recruited conditionally on being current injectors and relates to potential selection bias in favour of IDU with longer periods of injecting. There will also be right truncation in that some subjects have not yet ceased injecting.

**Figure 3:**
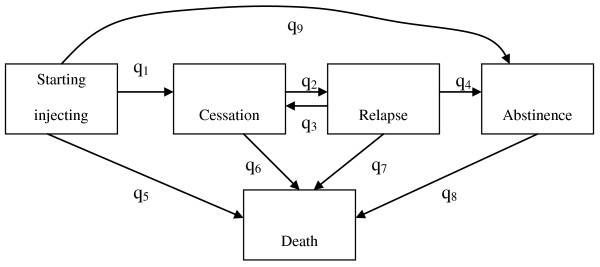
**Multi-state model of injecting history**. graphical depiction of the multi-state model of injecting history used in the analysis

We believe that the above challenges can be accommodated in a modelling framework such as that in figure 3 Here a multi-state model describes the injecting history in terms of five states: starting injecting; cessation of injecting; relapse into injecting; permanent abstinence from injecting; and death. The rates between the stages govern the average time subjects spend in each stage. Thus, q1 the rate of cessation for IDU that will relapse, and q9, the rate of cessation for people who become abstinent, together with q5 determine the length of time spent in first period of injecting. Analogously, the rates q2 (relapse), q3 (recovery in people that may later relapse), and q4 (abstinence), together with the death rates (q6, q7) determine the average time of injecting and non-injecting between injecting periods. This modelling approach is flexible and can allow rates to change with covariates (such as socio-demographic characteristics, age of onset, and exposure to treatment) and can accommodate partial observations and problem of left and right truncation [41,42].

## Conclusions

Injecting drug users recruited from a community setting can be successfully followed-up through interviews and record linkage. Injecting life course information collected will provide basis for analyses of injecting duration. Further comparisons between cases and controls will allow identification of possibly modifiable early life risk factors for drug injection and will also clarify the burden of disease associated with injection and the influence on this of different health and social interventions.

## Competing interests

The authors declare that they have no competing interests.

## Authors' contributions

JRR is Principle Investigator of the Edinburgh Addiction Study. JM, JRR and MH conceived the present follow up study and obtained funding. LC and JMcK undertook the fieldwork. MH and JM conceived the analytic strategy with JK and DD. LC undertook descriptive analyses with JK; JK and DD undertook other analyses including modeling. JM wrote the first draft of the paper with all authors contributing to the final draft. JRR is study guarantor.

## Pre-publication history

The pre-publication history for this paper can be accessed here:

http://www.biomedcentral.com/1471-2458/10/101/prepub

## Supplementary Material

Additional file 1 **Appendix 1**. the interview schedule used in the Edinburgh Addiction Cohort study Click here for file

Additional file 2 **Appendix 2**. the form used for recording of information extracted from primary care records Click here for file
