# Supplementary material for: The Edinburgh Addiction Cohort: recruitment and follow-up of a primary care based sample of injection drug users and non drug-injecting controls
Source: BMC Public Health. 2010 Feb 26;10:101. doi: 10.1186/1471-2458-10-101 (PMC2841670; doi:10.1186/1471-2458-10-101)
Supplement: Additional file 1 — Appendix 1. the interview schedule used in the Edinburgh Addiction Cohort study [file 1471-2458-10-101-S1.DOC]

### INTERVIEW SCHEDULE – EDINBURGH ADDICTION COHORT STUDY

*Instructions for interviewer:*

1. *Note significant life events on grid.*
2. *Complete sections on drug history, treatment history, criminal history, early life and health from questionnaire with reference to life grid to fix certain dates.*

| Surname | First name |
| --- | --- |
| Date of birth | Date of interview |
| Place of birth | Length of interview |
| Ethnicity | Place of interview |
| Gender | Interviewer |

### SECTION 1 – HISTORY OF DRUG USE

**I would like to start the interview by asking you about the times you injected and other past or current drug use.**

1. From the following list of drugs can you say if you have ever used them or are still doing so?

| ***Substance*** | ***Used in past (yes/no)*** | ***Inject (yes/no)***  ***If applicable*** | ***Age of first use*** | ***Used in the last 3 months (yes/no)*** |
| --- | --- | --- | --- | --- |
| Smoke tobacco |  |  |  |  |
| Alcohol |  |  |  |  |
| Heroin |  |  |  |  |
| Cannabis |  |  |  |  |
| Illicit benzodiazepines |  |  |  |  |
| Illicit methadone |  |  |  |  |
| Illicit dihydrocodeine |  |  |  |  |
| Other illicit opiate |  |  |  |  |
| Barbiturates |  |  |  |  |
| Amphetamine |  |  |  |  |
| Cocaine |  |  |  |  |
| Crack |  |  |  |  |
| LSD |  |  |  |  |
| Ecstacy |  |  |  |  |
| Temgesic |  |  |  |  |
| Other |  |  |  |  |

##### 2. How old were you when you started to inject?  

3. What drug(s) did you inject when you started?

|  |  |  |  |  |  |
| --- | --- | --- | --- | --- | --- |

##### 4. Who else was involved?

Friend/s  Partner 

Sibling/s 

Other family members (who?)

Other

**5. What happened to them?** [probe – when did they cease injecting, are they still injecting, alive – if known]

|  |
| --- |
|  |
|  |
|  |

6. Did you inject first time or did someone inject you?

|  |
| --- |
|  |

7. If someone else injected you, who was this?

|  |
| --- |
|  |

8. When did you start to inject yourself?

|  |
| --- |
|  |

9. When did you start to inject regularly?

|  |
| --- |
|  |

10. At the time when your injecting was at its most frequent how much did you use? (relates to main drug injected)

|  |
| --- |
|  |

11. Looking back over the years, can you remember times when you didn’t inject?

INTERVIEWER – come back to this and life grid when completing prison and treatment questions to see if periods of cessation correspond/overlap with periods of Rx and imprisonment? (use codes card for stopped/started)

| ***Year of first injecting*** | ***Inject***  ***this***  ***year***  ***(yes/no)*** | ***Cease injecting***  ***for 3 + months***  ***(yes/no)*** | Number of  times ceased for 3+ months? | ***If less 3 months how many days ceased?*** | ***If stopped***  ***why?*** | ***If started again***  ***why?*** | ***Can you estimate how many days you injected this year?*** | ***Can you estimate how many times per day you injected?*** |
| --- | --- | --- | --- | --- | --- | --- | --- | --- |
| 1960 |  |  |  |  |  |  |  |  |
| 1961 |  |  |  |  |  |  |  |  |
| 1962 |  |  |  |  |  |  |  |  |
| 1963 |  |  |  |  |  |  |  |  |
| 1964 |  |  |  |  |  |  |  |  |
| 1965 |  |  |  |  |  |  |  |  |
| 1966 |  |  |  |  |  |  |  |  |
| 1967 |  |  |  |  |  |  |  |  |
| 1968 |  |  |  |  |  |  |  |  |
| 1969 |  |  |  |  |  |  |  |  |
| 1970 |  |  |  |  |  |  |  |  |
| ***Year of first injecting*** | ***Inject***  ***this***  ***year***  ***(yes/no)*** | ***Cease injecting***  ***for 3 + months***  ***(yes/no)*** | Number of  times ceased for 3+ months? | ***If less 3 months how many days ceased?*** | ***If stopped why?*** | ***If started again why?*** | ***Can you estimate how many days you injected this year?*** | ***Can you estimate how many times per day you injected?*** |
| 1971 |  |  |  |  |  |  |  |  |
| 1972 |  |  |  |  |  |  |  |  |
| 1973 |  |  |  |  |  |  |  |  |
| 1974 |  |  |  |  |  |  |  |  |
| 1975 |  |  |  |  |  |  |  |  |
| 1976 |  |  |  |  |  |  |  |  |
| 1977 |  |  |  |  |  |  |  |  |
| 1978 |  |  |  |  |  |  |  |  |
| 1979 |  |  |  |  |  |  |  |  |
| 1980 |  |  |  |  |  |  |  |  |
| 1981 |  |  |  |  |  |  |  |  |
| 1982 |  |  |  |  |  |  |  |  |
| ***Year of first injecting*** | ***Inject***  ***this***  ***year***  ***(yes/no)*** | ***Cease injecting***  ***for 3 + months***  ***(yes/no)*** | Number of  times ceased for 3+ months? | ***If less 3 months how many days ceased?*** | ***If stopped why?*** | ***If started again why?*** | ***Can you estimate how many days you injected this year?*** | ***Can you estimate how many times per day you injected?*** |
| 1983 |  |  |  |  |  |  |  |  |
| 1984 |  |  |  |  |  |  |  |  |
| 1985 |  |  |  |  |  |  |  |  |
| 1986 |  |  |  |  |  |  |  |  |
| 1987 |  |  |  |  |  |  |  |  |
| 1988 |  |  |  |  |  |  |  |  |
| 1989 |  |  |  |  |  |  |  |  |
| 1990 |  |  |  |  |  |  |  |  |
| 1991 |  |  |  |  |  |  |  |  |
| 1992 |  |  |  |  |  |  |  |  |
| 1993 |  |  |  |  |  |  |  |  |
| 1994 |  |  |  |  |  |  |  |  |
| ***Year of first injecting*** | ***Inject***  ***this***  ***year***  ***(yes/no)*** | ***Cease injecting***  ***for 3 + months***  ***(yes/no)*** | Number of  times ceased for 3+ months? | ***If less 3 months how many days ceased?*** | ***If stopped why?*** | ***If started again why?*** | ***Can you estimate how many days you injected this year?*** | ***Can you estimate how many times per day you injected?*** |
| 1995 |  |  |  |  |  |  |  |  |
| 1996 |  |  |  |  |  |  |  |  |
| 1997 |  |  |  |  |  |  |  |  |
| 1998 |  |  |  |  |  |  |  |  |
| 1999 |  |  |  |  |  |  |  |  |
| 2000 |  |  |  |  |  |  |  |  |
| 2001 |  |  |  |  |  |  |  |  |
| 2002 |  |  |  |  |  |  |  |  |
| 2003 |  |  |  |  |  |  |  |  |
| 2004 |  |  |  |  |  |  |  |  |
| 2005 |  |  |  |  |  |  |  |  |
| 2006 |  |  |  |  |  |  |  |  |
| 2007 |  |  |  |  |  |  |  |  |

**12. During your injecting career what was the longest period you didn’t inject?**

### SECTION 2 – DRUG TREATMENT HISTORY

**13(a) When did you first present at Muirhouse Surgery and say you had a drug problem? (*Year/Age)***

**(b) Was this injecting?** Yes No 

**(c) If “No”, when was the first time you said you had a problem with injecting? (*Year/Age)***

**14. When did you first get any treatment for your drug problem? *(Year/Age)***

##### 15. Have you ever been prescribed any of the following treatments?

| ***Prescription drug*** | ***Prescribed (yes/no)*** | ***Age when started*** | ***If still on this prescription, how long has this been for?*** | ***If stopped, how long were you on it?*** |
| --- | --- | --- | --- | --- |
| Methadone |  |  |  |  |
| Dihydrocodeine |  |  |  |  |
| Other opiates (not for pain relief) |  |  |  |  |
| Benzodiazepines |  |  |  |  |
| Buprenorphine |  |  |  |  |

**16(a) Have you ever had detoxification (detox) treatment?** Yes No 

**(b) If “yes” where was this?**

In the community (GP supported)  Residential setting  At home (self-supported)  Prison 

(c) If “residential” how long did you stay for?

| Setting (name) | Year/Age | Length of time |
| --- | --- | --- |
|  |  |  |
|  |  |  |
|  |  |  |

##### 17(a) Have you been referred to specialist drug treatment services? Yes  No 

**(b) I**f “yes” please complete the following table:

| ***Age referred*** | ***Treatment received*** | | | |
| --- | --- | --- | --- | --- |
|  | ***Counselling*** | ***Maintenance prescription*** | ***Detox prescription*** | ***Other treatment (please specify)*** |
|  |  |  |  |  |
|  |  |  |  |  |
|  |  |  |  |  |
|  |  |  |  |  |
|  |  |  |  |  |

**18(a) While on treatment did you continue to inject?** Yes  No 

**(b) Use other drugs?** Yes  No 

**(c) If “yes” what were they?**

# SECTION 3 – EARLY AND PRESENT LIFE

#### 19. Who took care of you while you were growing up, during periods pre-school, primary school, secondary school? (Tick all that apply)

| ***Carer*** | ***Pre-school 1-5*** | ***Primary*** | ***Secondary*** |
| --- | --- | --- | --- |
| Both parents |  |  |  |
| Mother only |  |  |  |
| Father only |  |  |  |
| Mother and step father |  |  |  |
| Father and step mother |  |  |  |
| Grandparent(s) |  |  |  |
| In care |  |  |  |
| Adoptive/foster parents |  |  |  |
| Other relative – specify |  |  |  |
| Other guardian - specify |  |  |  |
| Other combination - specify |  |  |  |

#### 20. What was the main source of money/ household income? (Tick all that apply)

| ***Income*** | ***Pre-school 1-5*** | Primary | ***Secondary*** |
| --- | --- | --- | --- |
| Benefits |  |  |  |
| Job |  |  |  |
| Other (specify) |  |  |  |
| If Job – whose job? |  |  |  |
| What Job? |  |  |  |

#### 21. When you were growing up, were there any problems that you can remember? (Tick or complete all that apply)

| ***Problem*** | ***Pre-school***  ***1-5*** | ***Primary*** | ***Secondary*** |
| --- | --- | --- | --- |
| Have you had a serious illness or been seriously injured? |  |  |  |
| Has one of your family been seriously ill of injured? |  |  |  |
| Close friends or relatives been seriously ill or injured? |  |  |  |
| Member of family died (specify)? |  |  |  |
| Close relatives or friends died? |  |  |  |
| Parents divorce or separate? |  |  |  |
| Immediate family member subjected to form of serious abuse attack or threat? |  |  |  |
| Parents/carer unemployed? |  |  |  |
| Parents/carer made redundant? |  |  |  |
| Number of times had to move house (not by choice)? |  |  |  |
| Major financial difficulties e.g. debts difficulty paying bills? |  |  |  |
| Parents or family in contact with police or been in court? |  |  |  |
| Have you or immediate family been mugged or burgled? |  |  |  |
| Witness violence in family (from dad to mum, or other - specify)? |  |  |  |
| Witness violence from parent to sibling? |  |  |  |
| Experience violence from mum, dad or carer (or other family member – specify)? |  |  |  |
| Experience sexual abuse? |  |  |  |
| Any other problems that you remember? |  |  |  |

22(a) Did either of your parents (or person who had main care of you while growing up) do any of the following?

|  | *Yes/no* | *If “yes” who?* |
| --- | --- | --- |
| Smoke cigarettes |  |  |
| Have alcohol problems |  |  |
| Use drugs |  |  |

**(b) If your carer used drugs” did they inject?** Yes  No 

(c) If “yes” what did you feel about this?

|  |
| --- |
|  |
|  |

**23(a) Do you have any brothers and sisters (full)?** Yes  No 

#### (b) If “yes”, how many? 

(c) Other (e.g. from adopted family)

**24. How many schools did you attend? Primary School**  **Secondary School** 

**25(a) Were you ever excluded from school?** Yes  No 

**(b) If “yes”, how many times?** 

(c) Why?

|  |
| --- |

**26(a) Were you ever sent to see a psychologist or someone for problems at school?** Yes  No 

**(b) If “yes”, how many times?** 

***(c) Why?***

|  |
| --- |

27(a) Were you ever referred to social services or had a case conference/ family panel while growing up? Yes  No 

(b) If “yes”, how many times? 

***(c) Why?***

|  |
| --- |

**28(a) Were you ever arrested by police? Yes**  **No** 

**If “yes” how many times during primary school**  **secondary school** 

**(b)Age first arrest?**  **(c)Number of times been to court?**  **(d)Age first court appearance?** 

**29(a) Have you ever been in borstal/young offenders/List D school?** Yes  No 

(b) If “yes”, complete the following details:

| ***Type of institution*** | ***Year of entry*** | ***Age at entry*** | ***Time spent there*** | ***Drugs used*** | ***Inject Yes/No*** |
| --- | --- | --- | --- | --- | --- |
|  |  |  |  |  |  |
|  |  |  |  |  |  |
|  |  |  |  |  |  |

##### 30. What qualifications did you get at school or further education?

##### Number of standard grades 

##### Number of highers 

Further education qualifications

Other

**31. What age were you when you left school?** 

##### 32. What did you do when you left school?

Started work 

Went to college/university 

Government work opportunities scheme (If “yes” please specify) 

Nothing 

Other

###### 33. What is your current employment situation?

Never employed  Employed  Unemployed (1 year or longer)  Unable to work due to ill health (official)  Unemployed (less than a year) 

##### 34. List occupations held (first and last only)

| First |  | Last |  |
| --- | --- | --- | --- |

**35. What is your current main source of income?**

| Official paid employment |  |
| --- | --- |
| Unofficial paid employment |  |
| Unemployment benefit |  |
| Sickness benefit |  |
| Prostitution |  |
| Criminal activities (any type) |  |
| Family |  |
| Other (specify) |  |

36. Have you ever obtained income in any of the following ways?

| ***Method of obtaining income*** | ***yes/no*** | ***Number of times***  ***1 = <10; 2 = >or = 10 and <30; 3 = >or = 30 and <50; 4 = >or= 50 and < =100 ; 5 = hundreds; 6 = >1000*** |
| --- | --- | --- |
| Selling drugs |  |  |
| Begging |  |  |
| Handling stolen goods |  |  |
| Shoplifting |  |  |
| Housebreaking |  |  |
| Fraud/forgery |  |  |
| Prostitution |  |  |

37. Do either of the following financial situations apply to you currently or in the past?

|  | *Past (yes/no?)* | *Current (yes/no)* |
| --- | --- | --- |
| Debts which cause serious worry (legal) |  |  |
| Debts which cause serious worry (illegal) |  |  |

**38. Have you ever been in prison?** Yes  No  **If “yes”, interviewer please complete the following chart. Length = no. of days. Refer back to drug profile** **in q 11**

| **1971** | | **1972** | | **1973** | | **1974** | | **1975** | |
| --- | --- | --- | --- | --- | --- | --- | --- | --- | --- |
| Prison (Days) |  | Prison (Days) |  | Prison (Days) |  | Prison (Days) |  | Prison (Days) |  |
| Crime |  | Crime |  | Crime |  | Crime |  | Crime |  |
| Prescription drugs taken while in prison |  | Prescription drugs taken while in prison |  | Prescription drugs taken while in prison |  | Prescription drugs taken while in prison |  | Prescription drugs taken while in prison |  |
| Illicit drugs taken while in prison |  | Illicit drugs taken while in prison |  | Illicit drugs taken while in prison |  | Illicit drugs taken while in prison |  | Illicit drugs taken while in prison |  |
| Inject while in prison |  | Inject while in prison |  | Inject while in prison |  | Inject while in prison |  | Inject while in prison |  |
| **1976** | | **1977** | | **1978** | | **1979** | | **1980** | |
| Prison (Days) |  | Prison (Days) |  | Prison (Days) |  | Prison (Days) |  | Prison (Days) |  |
| Crime |  | Crime |  | Crime |  | Crime |  | Crime |  |
| Prescription drugs taken while in prison |  | Prescription drugs taken while in prison |  | Prescription drugs taken while in prison |  | Prescription drugs taken while in prison |  | Prescription drugs taken while in prison |  |
| Illicit drugs taken while in prison |  | Illicit drugs taken while in prison |  | Illicit drugs taken while in prison |  | Illicit drugs taken while in prison |  | Illicit drugs taken while in prison |  |
| Inject while in prison |  | Inject while in prison |  | Inject while in prison |  | Inject while in prison |  | Inject while in prison |  |

| **1981** | | **1982** | | **1983** | | **1984** | | **1985** | |
| --- | --- | --- | --- | --- | --- | --- | --- | --- | --- |
| Prison (Days) |  | Prison (Days) |  | Prison (Days) |  | Prison (Days) |  | Prison (Days) |  |
| Crime |  | Crime |  | Crime |  | Crime |  | Crime |  |
| Prescription drugs taken while in prison |  | Prescription drugs taken while in prison |  | Prescription drugs taken while in prison |  | Prescription drugs taken while in prison |  | Prescription drugs taken while in prison |  |
| Illicit drugs taken while in prison |  | Illicit drugs taken while in prison |  | Illicit drugs taken while in prison |  | Illicit drugs taken while in prison |  | Illicit drugs taken while in prison |  |
| Inject while in prison |  | Inject while in prison |  | Inject while in prison |  | Inject while in prison |  | Inject while in prison |  |
| **1986** | | **1987** | | **1988** | | **1989** | | **1990** | |
| Prison (Days) |  | Prison (Days) |  | Prison (Days) |  | Prison (Days) |  | Prison (Days) |  |
| Crime |  | Crime |  | Crime |  | Crime |  | Crime |  |
| Prescription drugs taken while in prison |  | Prescription drugs taken while in prison |  | Prescription drugs taken while in prison |  | Prescription drugs taken while in prison |  | Prescription drugs taken while in prison |  |
| Illicit drugs taken while in prison |  | Illicit drugs taken while in prison |  | Illicit drugs taken while in prison |  | Illicit drugs taken while in prison |  | Illicit drugs taken while in prison |  |
| Inject while in prison |  | Inject while in prison |  | Inject while in prison |  | Inject while in prison |  | Inject while in prison |  |
| **1991** | | **1992** | | **1993** | | **1994** | | **1995** | |
| Prison (Days) |  | Prison (Days) |  | Prison (Days) |  | Prison (Days) |  | Prison (Days) |  |
| Crime |  | Crime |  | Crime |  | Crime |  | Crime |  |
| Prescription drugs taken while in prison |  | Prescription drugs taken while in prison |  | Prescription drugs taken while in prison |  | Prescription drugs taken while in prison |  | Prescription drugs taken while in prison |  |
| Illicit drugs taken while in prison |  | Illicit drugs taken while in prison |  | Illicit drugs taken while in prison |  | Illicit drugs taken while in prison |  | Illicit drugs taken while in prison |  |
| Inject while in prison |  | Inject while in prison |  | Inject while in prison |  | Inject while in prison |  | Inject while in prison |  |
| **1996** | | **1997** | | **1998** | | **1999** | | **2000** | |
| Prison (Days) |  | Prison (Days) |  | Prison (Days) |  | Prison (Days) |  | Prison (Days) |  |
| Crime |  | Crime |  | Crime |  | Crime |  | Crime |  |
| Prescription drugs taken while in prison |  | Prescription drugs taken while in prison |  | Prescription drugs taken while in prison |  | Prescription drugs taken while in prison |  | Prescription drugs taken while in prison |  |
| Illicit drugs taken while in prison |  | Illicit drugs taken while in prison |  | Illicit drugs taken while in prison |  | Illicit drugs taken while in prison |  | Illicit drugs taken while in prison |  |
| Inject while in prison |  | Inject while in prison |  | Inject while in prison |  | Inject while in prison |  | Inject while in prison |  |
| **2001** | | **2002** | | **2003** | | **2004** | | **2005** | |
| Prison (Days) |  | Prison (Days) |  | Prison (Days) |  | Prison (Days) |  | Prison (Days) |  |
| Crime |  | Crime |  | Crime |  | Crime |  | Crime |  |
| Prescription drugs taken while in prison |  | Prescription drugs taken while in prison |  | Prescription drugs taken while in prison |  | Prescription drugs taken while in prison |  | Prescription drugs taken while in prison |  |
| Illicit drugs taken while in prison |  | Illicit drugs taken while in prison |  | Illicit drugs taken while in prison |  | Illicit drugs taken while in prison |  | Illicit drugs taken while in prison |  |
| Inject while in prison |  | Inject while in prison |  | Inject while in prison |  | Inject while in prison |  | Inject while in prison |  |
| **2006** | | **2007** | |  | | | | | |
| Prison (Days) |  | Prison (Days) |  |
| Crime |  | Crime |  |
| Prescription drugs taken while in prison |  | Prescription drugs taken while in prison |  |
| Illicit drugs taken while in prison |  | Illicit drugs taken while in prison |  |
| Inject while in prison |  | Inject while in prison |  |

#### 39. How would you describe your current martial status?

Married 

Single 

Widowed 

Separated 

Divorced 

Partner 

**40.** **How many relationships have you been involved in which have lasted longer than a year?** 

**41(a) Do you have any children?** Yes  No 

**(b) If “yes”, how many?** 

**(c) Are they from different relationships?** Yes  No 

**(d) From how many relationships?** 

#### 42. Have any of your children experienced any of the following situations?

Lived mainly with other parent Lived mainly with other relative 

Periods in care Fostered 

Adopted 

#### 43(a) Have you ever had any periods of homelessness greater than 2 weeks (when did not have your own roof over your head)? Yes  No 

(b) If “yes” complete the following:

| *Type of sleeping arrangements* | *Year/ age* | *Weeks/*  *Months* | *Year/ age* | *Weeks/*  *Months* | *Year/ age* | *Weeks/*  *Months* | *Total number of times* |
| --- | --- | --- | --- | --- | --- | --- | --- |
| Hostel/shelter |  |  |  |  |  |  |  |
| Sleeping rough |  |  |  |  |  |  |  |
| Sleeping in various friends’ houses |  |  |  |  |  |  |  |

**44. What type of accommodation do you live in at present?**

Rented 

Owned 

Other

**45. Since becoming an adult, can you estimate how many times you have changed accommodation? (this applies to accommodation you have stayed in for 6 months or more)** 

#### 46. Who do you currently live with?

| Spouse/partner |  |
| --- | --- |
| Alone |  |
| With sibling/s |  |
| With other relative/s |  |
| With friends |  |
| Alone with children |  |
| With spouse/partner and children |  |
| Parent/s |  |

**SECTION 4 - HEALTH**

##### 47(a) Have you ever overdosed and been seen by a doctor? Yes  No 

(b) If “yes” please complete the following table:

| ***Year/Age*** | ***Drugs involved (list)*** | ***Injecting involved***  ***(yes/no)*** | ***Hospital involved***  ***(yes/no)*** | ***Intentional***  ***(yes/no)*** |
| --- | --- | --- | --- | --- |
|  |  |  |  |  |
|  |  |  |  |  |
|  |  |  |  |  |

**48(a) Have you ever had a mental health diagnosis?** Yes  No 

(b) If “yes” please complete the following table:

| ***Diagnosis*** | ***Age*** |
| --- | --- |
|  |  |
|  |  |
|  |  |

**49(a) Have you ever had any suicide attempts?** Yes  No 

(b) If “yes” please complete the following table:

| ***Year/Age*** | ***Method*** | ***Year/Age*** | ***Method*** |
| --- | --- | --- | --- |
|  |  |  |  |
|  |  |  |  |
