# Supplementary material for: The Edinburgh Addiction Cohort: recruitment and follow-up of a primary care based sample of injection drug users and non drug-injecting controls
Source: BMC Public Health. 2010 Feb 26;10:101. doi: 10.1186/1471-2458-10-101 (PMC2841670; doi:10.1186/1471-2458-10-101)
Supplement: Additional file 2 — Appendix 2. the form used for recording of information extracted from primary care records [file 1471-2458-10-101-S2.DOC]

# EDINBURGH ADDICTION COHORT –

# CASE NOTE DATA EXTRACTION FORM

1. Demographics

| ***VARIABLE*** | ***RECORD*** | ***INSTRUCTION*** |
| --- | --- | --- |
| Patno |  |  |
| Surname |  |  |
| First name |  |  |
| Date of birth |  |  |
| Gender |  |  |
| Ethnic group |  |  |
| Current address |  |  |
| City |  |  |
| Postcode |  |  |
| Marital status |  | *1 = married, 2 = single, 3 = partner, 4 = widowed, 5 = separated, 6 = divorced* |
| MMG reg year |  | *year first registered at practice* |
| Year left MMG |  | *for those who have left MMG only* |

2. Background problems/education/employment/prison

| ***VARIABLE*** | ***RECORD*** | | ***INSTRUCTION*** |
| --- | --- | --- | --- |
| Case report/refer |  | | *any cases of referral/case reports during childhood;yes/no* |
| Problem/s |  | | *summarise family/child problem* |
| Employment status/type of income |  |  | *1 = never employed; 2 = unemployed >=1year;3 = unemployed < 1 year; 4 = currently employed; 5 = medically unfit for work; also note type of income e.g. DLA; income support etc.* |
| Prison | yes/no | | *ever noted as having been in prison; yes/no; record dates when noted as in prison* |
| Prison year (1) |  | Prison year (11) |  |
| Prison year (2) |  | Prison year (12) |  |
| Prison year (3) |  | Prison year (13) |  |
| Prison year (4) |  | Prison year (14) |  |
| Prison year (5) |  | Prison year (15) |  |
| Prison year (6) |  | Prison year (16) |  |
| Prison year (7) |  | Prison year (17) |  |
| Prison year (8) |  | Prison year (18) |  |
| Prison year (9) |  | Prison year (19) |  |
| Prison year (10) |  | Prison year (20) |  |
| Total prison sentences |  | | count |

1. Illicit drug use

| ***VARIABLE*** | ***RECORD*** | INSTRUCTION |
| --- | --- | --- |
| SMR date |  | *Date SMR form completed, if available* |
| First injected |  | *first noted injection date* |
| MMG inj date |  | *first presented at MMG with injecting problem* |
| Other surgery inj date |  | *for those offlist, record when presented at other surgery* |
| Drugs injected |  | *list any drugs injected* |
| Injecting (1) |  | *noted as injecting on this date* |
| Injecting (2) |  | *noted as injecting on this date* |
| Injecting (3) |  | *noted as injecting on this date* |
| Injecting (4) |  | *noted as injecting on this date* |
| Injecting (5) |  | *noted as injecting on this date* |
| Injecting (6) |  | *noted as injecting on this date* |
| Not injecting (1) |  | *note date when not injecting* |
| Not injecting (2) |  | *note date when not injecting* |
| Not injecting (3) |  | *note date when not injecting* |
| Not injecting (4) |  | *note date when not injecting* |
| Not injecting (5) |  | *note date when not injecting* |
| Not injecting (6) |  | *note date when not injecting* |
| Last injected |  | *last noted date of injecting* |

4. Alcohol/tobacco

| ***VARIABLE*** | ***RECORD*** | INSTRUCTION |
| --- | --- | --- |
| Smoking status |  | *1 = current; 2 = ex; 3 = never; 4 = don't know* |
| Smoker (current) date |  | *date (as current as possible)* |
| Smoker (current) |  | *number of cigarettes per day; 0 = no data available* |
| Alcohol problems |  | *alcohol problems mentioned in notes; yes/no, 0 = no data available* |
| Referred alcohol |  | *ever referred to alcohol problems clinic; yes/no* |
| Referred date (1) |  | *date of referral* |
| Attend (1) |  | *did patient attend? yes/no* |
| Referred date (2) |  | *date of referral* |
| Attend (2) |  | *did patient attend? yes/no* |
| Referred date (3) |  | *date of referral* |
| Attend (3) |  | *did patient attend? yes/no* |
| Total referrals |  | *Total number of referrals made* |
| Total attended |  | *Total number of referrals attended* |

5. Substitute prescribing/treatment exposure; specialist drug treatment

| ***VARIABLE*** | ***RECORD*** | INSTRUCTION | |
| --- | --- | --- | --- |
| Methadone |  | *ever prescribed; yes/no* | |
| 1st methadone script starts |  | *date; 0 = no details available in notes* | |
| 1st methadone script ends |  | *date; 0 = no details available in notes* | |
| 2^nd^ methadone script starts |  | *date; 0 = no details available in notes* | |
| 2^nd^ methadone script ends |  | *date; 0 = no details available in notes* | |
| 3^rd^ methadone script starts |  | *date; 0 = no details available in notes* | |
| 3^rd^ methadone script ends |  | *date; 0 = no details available in notes* | |
| Current methadone script date |  | *date; 0 = no longer on methadone* | |
| Total methadone scripts |  | *frequency of periods on methadone script* | |
| Months methadone |  | *total number of months on methadone* | |
| Df118s |  | *ever prescribed; yes/no* | |
| 1st df118 script starts |  | *date; 0 = no details available in notes* | |
| 1st df118 script ends |  | *date; 0 = no details available in notes* | |
| 2^nd^ df118 script starts |  | *date; 0 = no details available in notes* | |
| 2^nd^ df118 script ends |  | *date; 0 = no details available in notes* | |
| 3^rd^ df118 script starts |  | *date; 0 = no details available in notes* | |
| 3^rd^ df118 script ends |  | *date; 0 = no details available in notes* | |
| Current df118 script date |  | *date; 0 = no longer on methadone* | |
| Total df118 scripts |  | *frequency of periods on df118 script* | |
| Months df118 |  | *total number of months on df118* | |
| Other opiates (not for pain relief) |  | *ever prescribed; yes/no* |  |
| 1st other opiates script starts |  | *date; 0 = no details available in notes* |  |
| 1st other opiates script ends |  | *date; 0 = no details available in notes* |  |
| 2^nd^ other opiates script starts |  | *date; 0 = no details available in notes* |  |
| 2^nd^ other opiates script ends |  | *date; 0 = no details available in notes* |  |
| 3^rd^ other opiates script starts |  | *date; 0 = no details available in notes* |  |
| 3^rd^ other opiates script ends |  | *date; 0 = no details available in notes* |  |
| Current other opiates script date |  | *date; 0 = no longer on other opiates* |  |
| Total other opiates scripts |  | *frequency of periods on other opiates script* |  |
| Months other opiates |  | *total number of months on other opiates* |  |
| Benzos |  | *ever prescribed; yes/no* |  |
| 1st benzos script starts |  | *date; 0 = no details available in notes* |  |
| 1st benzos script ends |  | *date; 0 = no details available in notes* |  |
| 2^nd^ benzos script starts |  | *date; 0 = no details available in notes* |  |
| 2^nd^ benzos script ends |  | *date; 0 = no details available in notes* |  |
| 3^rd^ benzos script starts |  | *date; 0 = no details available in notes* |  |
| 3^rd^ benzos script ends |  | *date; 0 = no details available in notes* |  |
| Current benzos script date |  | *date; 0 = no longer on benzos* |  |
| Total benzos scripts |  | *frequency of periods on benzos script* |  |
| Months benzos |  | *total number of months on benzos* |  |
| Buprenorphine |  | *ever prescribed; yes/no* |  |
| 1st buprenorphine script starts |  | *date; 0 = no details available in notes* |  |
| 1st buprenorphine script ends |  | *date; 0 = no details available in notes* |  |
| 2^nd^ buprenorphine script starts |  | *date; 0 = no details available in notes* |  |
| 2^nd^ buprenorphine script ends |  | *date; 0 = no details available in notes* |  |
| 3^rd^ buprenorphine script starts |  | *date; 0 = no details available in notes* |  |
| 3^rd^ buprenorphine script ends |  | *date; 0 = no details available in notes* |  |
| Current buprenorphine script date |  | *date; 0 = no longer on buprenorphine* |  |
| Total buprenorphine scripts |  | *frequency of periods on buprenorphine script* |  |
| Months buprenorphine |  | *total number of months on buprenorphine* |  |

6. Specialist drug treatment service

| ***VARIABLE*** | ***RECORD*** | INSTRUCTION |
| --- | --- | --- |
| Ever referred to specialist drug treatment service |  | *yes/no* |
| 1st referral date |  | *initial consultation to initiate a period of treatment;0 = no data available in notes* |
| Attend 1st referral |  | *yes/no* |
| Referral date (2) |  | *2nd consultation to initiate a period of treatment* |
| Attend (2) |  | *yes/no* |
| Referral date (3) |  | *3rd consultation to initiate a period of treatment* |
| Attend (3) |  | *yes/no* |
| Referral date (4) |  | *4th consultation to initiate a period of treatment* |
| Attend (4) |  | *yes/no* |
| Referral date (5) |  | *5th consultation to initiate a period of treatment* |
| Attend (5) |  | *yes/no* |
| Referral date (6) |  | *6^th^ consultation to initiate a period of treatment* |
| Attend (6) |  | *yes/no* |
| Referral date (7) |  | *7th consultation to initiate a period of treatment* |
| Attend (7) |  | *yes/no* |
| Referral date (8) |  | *8th consultation to initiate a period of treatment* |
| Attend (8) |  | *yes/no* |
| Referral date (9) |  | *9th consultation to initiate a period of treatment* |
| Attend (9) |  | *yes/no* |
| Referral date (10) |  | *10th consultation to initiate a period of treatment* |
| Attend (10) |  | *yes/no* |
| Total referrals made |  | *total number of referrals made* |
| Total referrals attended |  | *total number of referrals attended* |

7. Medical history

| ***VARIABLE*** | ***RECORD*** | | | INSTRUCTION |
| --- | --- | --- | --- | --- |
| Serious mental illness |  |  |  | *Diagnosis: 1 = psychosis; 2 = manic depression; 3 = affective disorder; 0 = no diagnosis* |
| SMI date |  |  |  | *date of smi diagnosis* |
| SMI refer |  |  |  | *ever referred to specialist psychiatric service for smi; yes/no* |
| SMI attend |  |  |  | *did patient attend consultation?;yes/no* |
| Other mental health |  |  |  | *Diagnosis: 1 = depression and neuroses; 0 = no diagnosis* |
| OMH date |  |  |  | *date of omh diagnosis* |
| OMH refer |  |  |  | *ever referred to specialist psychiatric service for omh; yes/no* |
| OMH attend |  |  |  | *did patient attend consultation?;yes/no* |
| Parasuicide/self-harm (1) |  | | | *episode resulting in patient attending for treatment; date (1)* |
| Parasuicide/self-harm (2) |  | | | *episode resulting in patient attending for treatment; date (2)* |
| Parasuicide/self-harm (3) |  | | | *episode resulting in patient attending for treatment; date (3)* |
| Overdose (1) |  | | | *accidental/poisoning resulting in patient attending for treatment; date (1)* |
| Overdose (2) |  | | | *accidental/poisoning resulting in patient attending for treatment; date (2)* |
| Overdose (3) |  | | | *accidental/poisoning resulting in patient attending for treatment; date (3)* |

| HIV negative |  | *last negative test* |
| --- | --- | --- |
| HIV positive |  | *first positive test* |
| Hepatitis C positive |  | *first positive test* |
| Positive Hep C PCR |  | *positive Hep C PCR tests* |
| Hepatitis C negative |  | *last negative test* |
| Last negative Hep C PCR |  | *last negative Hep C PCR* |
| Hepatitis B core antibody negative |  | *date* |
| Hepatitis B core antibody positive |  | *date* |
| Hepatitis B surface antigen positive |  | *date* |
| Hepatitis B surface antigen negative |  | *date* |
| Hepatitis B vacc |  | *has patient been vaccinated for Hep B? yes/no;* |
| Significant illness 1 |  | *major diagnosis, 0 = no significant illnesses* |
| Significant illness 1 (date) |  | *date of diagnosis* |
| Significant illness 2 |  | *major diagnosis, 0 = no significant illnesses* |
| Significant illness 2 (date) |  | *date of diagnosis* |
| Significant illness 3 |  | *major diagnosis, 0 = no significant illnesses* |
| Significant illness 3 (date) |  | *date of diagnosis* |
| Significant illness 4 |  | *major diagnosis, 0 = no significant illnesses* |
| Significant illness 4 (date) |  | *date of diagnosis* |
| Significant illness 5 |  | *major diagnosis, 0 = no significant illnesses* |
| Significant illness 5 (date) |  | *date of diagnosis* |
